# Understanding the Impact of Synthetic Hematocrit Levels and Biomimetic Channel Widths on Bubble Parameters in Vascular Systems on a Chip

**DOI:** 10.3390/biomimetics10020098

**Published:** 2025-02-09

**Authors:** Karine Baassiri, Dan V. Nicolau

**Affiliations:** Department of Bioengineering, Faculty of Engineering, McGill University, Montreal, QC H3A 0E9, Canada; karine.baassiri@mail.mcgill.ca

**Keywords:** gas embolism, microfluidics, PDMS, hematocrit, microvasculature, bubbles

## Abstract

Gas embolism is a rare but life-threatening process characterized by the presence of gas bubbles in the venous or arterial systems. These bubbles, if sufficiently large or numerous, can block the delivery of oxygen to critical organs, in particular the brain, and subsequently they can trigger a cascade of adverse biochemical reactions with severe medical outcomes. Despite its critical nature, gas embolism remains poorly understood, necessitating extensive investigation, particularly regarding its manifestations in the human body and its modulation by various biological conditions. However, given its elusive nature, as well as potential lethality, gas embolism is extremely difficult to study in vivo, and nearly impossible to be the subject of clinical trials. To this end, we developed a microfluidic device designed to study in vitro the impact of blood properties and vascular geometries on the formation and evolution of gas bubbles. The system features a biomimetic vascular channel surrounded by two pressure chambers, which induce the genesis of bubbles under varying circumstances. The bubble parameters were correlated with different input parameters, i.e., channel widths, wall thicknesses, viscosities of the artificial blood, and pressure levels. Smaller channel widths and higher equivalent hematocrit concentrations in synthetic blood solutions increased the nucleation density and bubble generation frequencies. Small channel widths were also more prone to bubble formation, with implications for the vulnerability of vascular walls, leading to increased risks of damage or compromise to the integrity of the blood vessels. Larger channel widths, along with higher equivalent hematocrit concentrations, translated into larger bubble volumes and decreased bubble velocities, leading to an increased risk of bubble immobilization within the blood vessels. This biomimetic approach provides insights into the impact of patient history and biological factors on the incidence and progression of gas embolism. Medical conditions, such as anemia, along with anatomical features related to age and sex—such as smaller blood vessels in women and children or larger vascular widths in adult men—affect the susceptibility to the initiation and progression of gas embolism, explored here in vitro through the development of a controlled, physiological-like environment. The analysis of the videos that recorded gas embolism events in vitro for systems where pressure is applied laterally on the microvasculature with thin walls, i.e., 50 μm or less, suggests that the mechanism of gas transfer for the pressure area to the blood is based on percolation, rather than diffusion. These findings highlight the importance of personalized approaches in the management and prevention of gas embolism.

## 1. Introduction

Vascular gas embolism, first reported in the late 19^th^ century [1], is characterized by the presence of an abnormally high number or volume of gas bubbles in the venous or arterial circulation [2]. This process can lead to the partial or complete blockage of blood flow, and—consequently—the oxygen starvation of essential organs, especially the brain, followed by a wide range of circulatory, cardiovascular, and neurological complications, potentially causing death [3]. Gas bubbles enter or form in the vascular system due to abrupt pressure variations surrounding the human body [4], direct introduction during medical procedures [5], trauma to gas-containing organs [6], lung over-expansion injury [6], or leakage from the venous to the arterial circulation [7]. The reported incidence rates of gas embolism vary widely due to the lack of specificity in its signs and symptoms, leading to difficulty in achieving a clear and accurate diagnosis [8]. For instance, these rates were reported to range from 0.2% for significant gas embolism [9] to as high as 50% for venous gas embolism [10]. On the other hand, in medical settings, the occurrence of gas embolism was reported to be 2.65 cases out of 100,000 hospitalizations [11]. Whatever the case, the actual rate of gas embolism is progressively increasing due to the growing number of invasive medical procedures [12]. In severe cases of gas embolism, the mortality rates were reported as 16% at hospital discharge and 21% within the following year [13], with severe neurological sequelae recorded as high as 35% [14]. The patient’s medical history is currently the most indicative factor in determining not only the risk, but also the progression and behavior of gas embolism [8]. However, given its elusive nature, as well as potential lethality, gas embolism is extremely difficult to study in vivo, and nearly impossible to be the subject of clinical trials. Therefore, in vitro investigations of the genesis and pathophysiology of vascular gas embolism can provide insights into its fundamental origins and mechanisms, ultimately improving diagnostic techniques, preventive measures, and medical treatments.

Microfluidics-based biomimetic systems were a suitable platform [15,16,17,18,19,20] for replicating gas embolism, due to their ability to simulate a controlled microenvironment and provide real-time monitoring of disease initiation and progression. This approach enables the use of microfluidic devices as vascular systems on a chip, ranging from diagnostics to complex organoids-on-a-chip that can mimic real-life systems and organs, such as the lungs [21]. Moreover, polydimethylsiloxane-based channels offer the ability to mimic blood vessels and biological tissue [22], facilitating the study of gas bubble formation at the onset of different nucleation sites by simulating sudden pressure variations, both globally and locally. Hydrodynamic techniques have been extensively researched and utilized for the creation of gaseous bubbles in microfluidic channels containing liquid solutions [23]. Several research efforts investigated the behavior, evolution, and biological effects of artificially induced bubbles in microfluidic devices [16,17,18,19,20]. However, the origins of gas embolism in biomimetic systems, with real-time observation and imaging of bubble genesis, triggered by global and local pressure applications, were never attempted.

The present work replicates gas embolism in vitro to investigate its origins and evolution mechanisms, through the study and quantification of bubble generation. By introducing microfluidic devices as vascular systems on a chip, our research objectives were twofold: first, to use microfluidic platforms to replicate gas embolism by exposing a central vascular channel to side local pressures; and second, to distinguish microbubble characteristics, such as volume, frequency, and velocity, based on rheological properties (viscosity and flow rate of the synthetic blood solution) and biomimetic network geometries (channel widths and wall thicknesses). The goal of this study is to ultimately contribute to the development of better diagnostic, preventive, and therapeutic strategies for gas embolism with personalized approaches.

## 2. Materials and Methods

The origins of intravascular bubble formation were explored in a controllable environment, mimicking the stress-induced responses of human blood vessels, upon exposure to increasing levels of pressure, at the microscopic scale.

**Figure 1:**
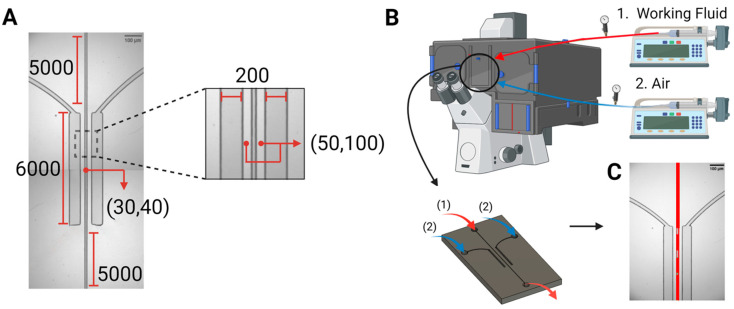
Device design and experimental setup. (**A**) The microfluidic device, i.e., the bilateral pressure chambers, consisted of a central vascular channel with a width of either 30 µm or 40 µm, surrounded by 200 μm wide pressure chambers on both sides. Dimensions are given in µm. (**B**). The microfluidic devices were placed in the microscope system, with inlets connected to two syringe pumps for the air stream (blue) and working fluids (red, taken from a reservoir at atmospheric conditions), and an outlet for draining the circulated working fluids (red, back into atmospheric conditions). (**C**). Patterns of bubble formation, resulting from pressure application on the vascular channel, via air injection into the side pressure chambers.

***Device design and fabrication.*** The microfluidic system comprised a central channel with widths of 30 μm, or alternatively 40 μm, simulating small arterioles and venules. This central channel hosting the flow of synthetic blood was surrounded by two chambers comprising pressured air, which spanned the entire length of the channel, i.e., 6 mm, on both sides, at a separation of 50 μm, or alternatively 100 μm. Figure 1A outlines the dimensions and features of the microfluidic structure.

After designing device layouts and printing the respective photomasks, the microfluidic devices were fabricated following two processing steps: fabrication of master structures by standard SU-8 photolithography, followed by polymer replica molding through soft lithography, using polydimethylsiloxane (PDMS) [24,25,26], to obtain the final chips. The optimum fabrication conditions required to achieve high dimensional accuracy when replicating the designed structures were established as follows: a spin coating speed of 4000 rpm; soft baking conditions of 3 min at 65 °C followed by 6 min at 95 °C; 55 s of ultraviolet exposure; and hard baking conditions of 2 min at 65 °C then 7 min at 95 °C. After replication, the devices were cured and treated with oxygen plasma before being sealed onto clean glass slides.

***Preparation of working fluid.*** The blood-mimicking solutions were prepared by mixing distilled water, glycerin, and Xanthan gum [27,28] on a hot plate at 35 °C to ensure optimal solubility and homogenization of the components. Simultaneously, the solution was subjected to continuous stirring using a magnetic stirrer at a consistent speed for 15 min. This ensured thorough dispersion of Xanthan gum, preventing clumping and facilitating the formation of a homogenous and stable solution. Two working fluids mimicked the rheological properties of anemic human blood, i.e., 20% hematocrit level, and healthy human blood, i.e., 46% hematocrit level, in terms of viscosity (typically between 3.5 and 5.5 cP [29]) and surface tension (usually in the range of 53 to 58 mN/m [28]). Although PDMS channels allow direct visualization of blood flow, a fluorescent tracer, namely fluorescein sodium salt, was incorporated into the blood formulations at a volume of 1 mL from a 2% stock solution for 50 mL of each working fluid. This guaranteed fast, easy, and high-resolution imaging of gas bubble formation and movement in microfluidic channels, without affecting the important characteristics of the blood-mimicking fluid.

***Experimental setup.*** The experimental setup (Figure 1B) consisted of an inverted confocal microscope (Olympus IX83 fluorescence microscope, Olympus Corporation, Tokyo, Japan), two programmable syringe pumps, i.e., Pump 11 Elite Syringe Pumps, Harvard Apparatus (Holliston, MA, USA) pumping liquid, and a BD Plastipak™ (Franklin Lakes, NJ, USA) syringe pumping air. Using the first syringe pump, the working fluid was injected, from a reservoir at atmospheric conditions, into the main channel at flow rates similar to real blood flow, i.e., 20 µL/h and 35 µL/h for the 30 µm and 40 µm widths, respectively [30,31]. The second pump was used to exert varying levels of pressure on the main channel, by injecting 1 to 20 mL of air into the bilateral pressure chambers at 100 mL/h. To calibrate the pressure drop in the central channel with fluid flow, and the pressure achieved in the lateral pressure chambers, control experiments were run with the microfluidics systems assisted by manometers (Bidirectional Microfluidic Flow Sensor, Fluigent, Paris, France).

***Image acquisition and analysis.*** The microfluidic devices were fixed on the microscope table, allowing high-speed imaging at sub-millimeter resolutions, using a 4X Uplano magnifying objective. The still images and movies were acquired at a frame rate of 50 frames per second (fps) using a high-speed camera (C11440-42U30, Hamamatsu Photonics K.K., Shizuoka, Japan). All acquired data were analyzed using the software ImageJ V1.53 (FIJI) based on variations in the fluorescence intensity. The output parameters related to bubble generation in the microchannels—including the onset (frame number at which bubbles began to emerge), nucleation density (sites of bubble formation per mm), generation frequency (number of bubbles per frame), mean velocity (distance over which bubbles travel per frame), and bubble volume (width × length × height)—were correlated with channel width, equivalent hematocrit concentration, gas transport distance, and applied pressure.

Aside from the analysis of still images, the dynamics of the generation of bubbles, modulated by the applied pressure for 50 μm separation, were observed in systems comparing the effect of two viscosities, i.e., 20% and 46% hematocrit concentrations in channels with 30 μm widths, and the effect of two channel widths, i.e., 30 μm and 40 μm, for blood with 20% hematocrit concentrations. The results are presented in Supplementary Information Movie S1 (30 μm/20% hematocrit), Movie S2 (30 μm/46% hematocrit), and Movie S3 (40 μm/20% hematocrit).

***Calculation of applied pressures on the mimicked blood vessel***. The local pressures applied sideways on the vascular channel were calculated based on the volume of injected air into the side pressure chambers. According to Boyle’s law, the product of the pressure and volume (P_1_V_1_) at atmospheric conditions must be equal to the resulting pressure and volume after air injection (P_2_V_2_). The initial pressure was assumed as 760 mmHg, and the initial volume was the designed separation distance of 50 µm × the height of the channel based on the spin coating conditions 40 µm × the designed length of the channel 6000 µm. The compressed volume of the interface between the two pressure chambers and the vascular channel was calculated after measuring the new separation distance using ImageJ (FIJI). Finally, the applied pressure on the vascular walls was calculated as P_2_ = (P_1_V_1_)/V_2_. Control experiments compared the calculated pressures in the lateral chambers with those measured using a manometer, installed immediately after the air pump. This comparison showed that the calculations were accurate (measured pressure = 0.9724 calculated pressure in the chambers, R² = 0.9983).

The measurements and the estimation of the pressure drop in the fluid phase used a similar methodology. The pressure was measured at the exit from the fluid pump—the exit from the microfluidic system being at atmospheric pressure. Using the Hagen–Poiseuille equation, the pressure drop was then estimated for the full circuit and for the region of interest, i.e., the central channel guarded by pressure chambers for a 6 mm long distance.

## 3. Results

***Mimicry of the microvasculature.*** Microfluidic devices were designed and fabricated as vascular systems on a chip made of PDMS, with bilateral pressure chambers, to mimic possible situations of gas embolism at the microscopic scale [24,25,26]. One possible natural system to be mimicked is the pulmonary alveolae, which has a total area in the range of 0.123 to 0.785 mm^2^, leading to an average area of ~0.455 mm^2^ that is exposed to gas transfer. By comparison, the central channel in the microfluidic devices, with a length of 6 mm and a height of 40 µm, presents an area for gas transfer (facing the parallel pressure chambers) of 0.48 mm^2^. Another possible real system to be mimicked is the exposure of microvasculature to pressured gases during surgery, e.g., laparoscopy (the microvasculature is typically located within the organs being irrigated).

The devices were placed in the microscope system, with inlets connected to two syringe pumps for the air stream (blue) and working fluids (red)**.** Two types of blood-equivalent solutions were injected into the channels at realistic flow rates, mimicking the circulatory system [27,28]. The rheological properties of the working fluid, widths of the vascular channel, and thicknesses of the vascular walls were evaluated in the central channel, which was undergoing uniform pressure variations along its entire length. This is particularly important because the onset of gas embolism is sudden and has never been observed and recorded in close to real-life scenarios, and it is, at least in the first instance, a predominantly physical phenomenon that requires precise control and observation of fluid behavior. The values of the pressure drop for these two types of fluids, in two channel widths, and for the full length of the microfluidic system, or the 6 mm length guarded by pressure chambers, respectively, are presented in Table 1.

### 3.1. Selection of Materials and Fluids Mimicking the Biological System

***Vascular systems on a chip.*** Polydimethylsiloxane (PDMS) was selected for fabricating the biomimetic devices, due to its biocompatibility, gas diffusivity, optical transparency, ease of replication, precise microfabrication [32], and inherent properties that are amenable to mimicking blood vessels [33,34]. More specifically, the Young’s Modulus of PDMS is close to the measured values in human vessels, reported to fall within the range of 0.05 to 2 MPa [35], but possibly going up to 5 MPa [36]. By comparison, the Young’s modulus for biological materials ranges from 0.05 to 2 MPa for the walls of the human veins [37], 0.04 to 2 MPa for abdominal aorta sections, 0.05 to 1.45 MPa for iliac arteries [38], and up to 5 MPa for cerebral arteries [36].

The diffusion coefficient of air in PDMS (10:1 mixing ratio) at 35 °C is between 3.21 × 10^−5^ cm^2^/s [39] and 3.40 × 10^−5^ cm^2^/s [40,41]. By comparison, there is a much larger variability in air diffusivity in human arteries, ranging from approximately 1 × 10^−5^ cm^2^/s [42] to 1.39 × 10^−5^ cm^2^/s [43]. However, in hamster retractor muscles comprising much finer blood vessels than human arteries, the diffusivity of air was reported as 2.42 × 10^−5^ cm^2^/s at 37 °C [42]. Consequently, for the even finer blood vessels considered in this study, PDMS can reasonably be considered as a material that mimics the diffusional properties of blood vessel walls.

***Synthetic blood solution.*** The working fluid must present rheological properties similar to those of real blood, including dynamic viscosity, shear-thinning behavior, and refractive index. Accordingly, two blood-mimicking solutions were prepared from a base mixture of 60% distilled water and 40% glycerin. Distilled water served as the primary solvent, while glycerin was incorporated to adjust the density and enhance the viscosity of the fluid. Xanthan gum, a high-molecular-weight polysaccharide, was subsequently added, at either 0.0075% or 0.04%, to emulate the non-Newtonian shear-thinning behavior of human blood at respective hematocrit concentrations of 20%, and 46%, representing mimicked blood from patients with anemia and normal individuals, respectively [44].

### 3.2. Proximity of the Application of Pressure

**Figure 2:**
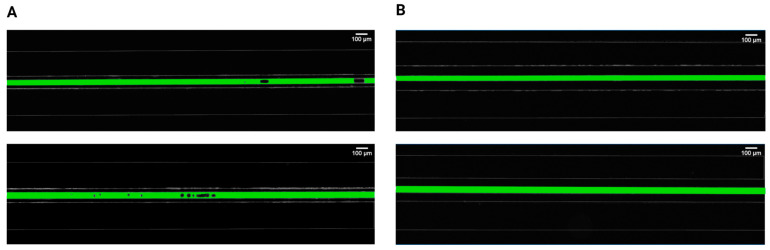
Comparison of intravascular bubble formation in 30 μm (first row) and 40 μm (second row) channels, surrounded by bilateral pressure chambers at distances of (**A**) 50 μm and (**B**) 100 μm. The injected air volume was maintained constant at 14 mL with a volumetric flow rate of 100 mL/h.

Initially, two separations between the pressured gas and the mimicked blood were evaluated and compared to determine the relationship between gas bubble formation and the proximity of applied pressures. It was found that bubble generation cannot occur for separations of 100 μm, irrespective of the injected air volume and flow rate (Figure 2). However, bubbles steadily emerged for the 50 μm separation, in both 30 μm and 40 μm channel widths, with different parameters related to bubble generation and dynamic behavior. This observation is consistent with earlier studies [19,45] investigating the gas diffusivity of PDMS that emphasized the significance of membrane thickness in diffusion-driven processes. These findings stated that for efficient gas diffusion, the thickness of PDMS membranes should ideally be around 50 μm, as thicker membranes were impeding pressure-driven processes. In an anatomical context, the majority of blood vessels are situated within the first 100 μm from airways [46,47], which makes our biomimetic microfluidic system relevant for the study of gas embolism in vitro.

After setting the separation at 50 μm, the generation of bubbles in biomimetic vascular channels was evaluated against the equivalent hematocrit concentrations, and vascular channel widths, following uniform exposure to different pressure levels. These pressures were achieved and maintained via the introduction of two air streams in both pressure chambers, at total volumes of injected air ranging between 1 and 20 mL, thereby controlling the extent of compression on the polymer and the neighboring channel.

### 3.3. Characteristics of the Generation of Bubbles

**Figure 3:**
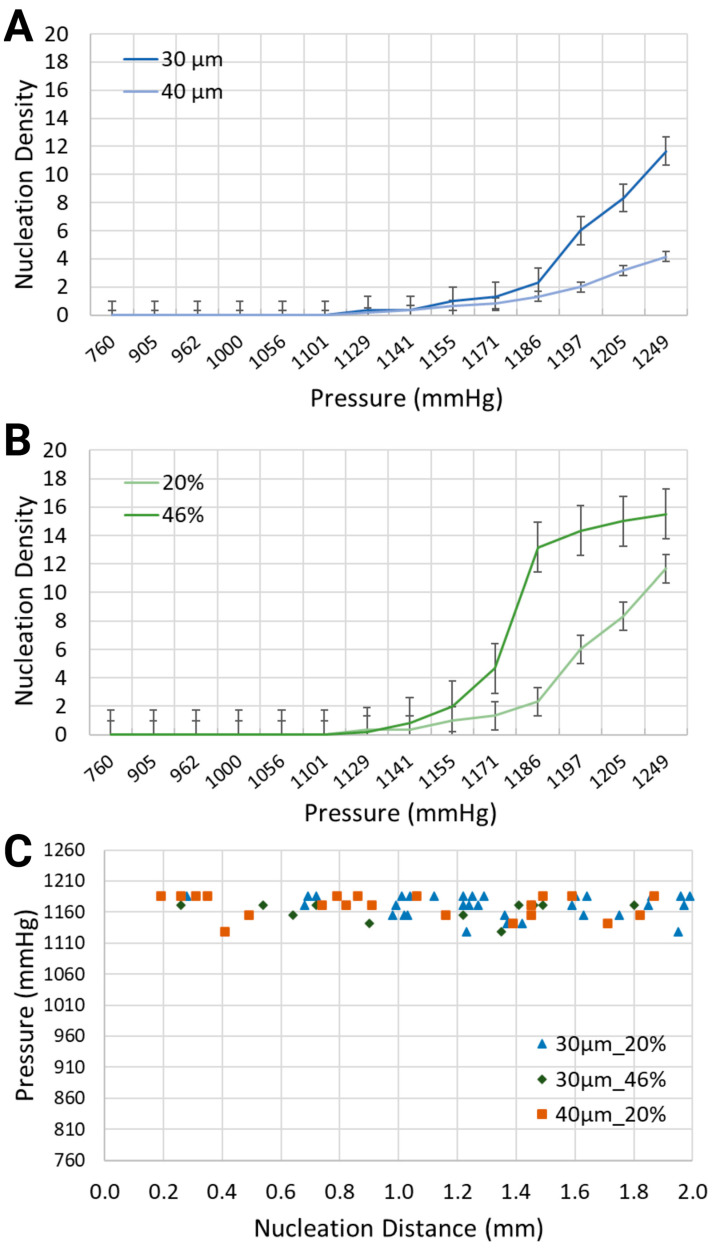
Nucleation density (nucleation sites/mm) for (**A**) two channel widths, i.e., 30 μm and 40 μm, containing a synthetic blood solution at 20% hematocrit level, and (**B**) the same channel width, i.e., 30 μm, containing two different synthetic blood solutions, at 20% and 46% hematocrit levels. (**C**) Location of nucleation sites with respect to the inlet of the synthetic blood solutions.

**Figure 4:**
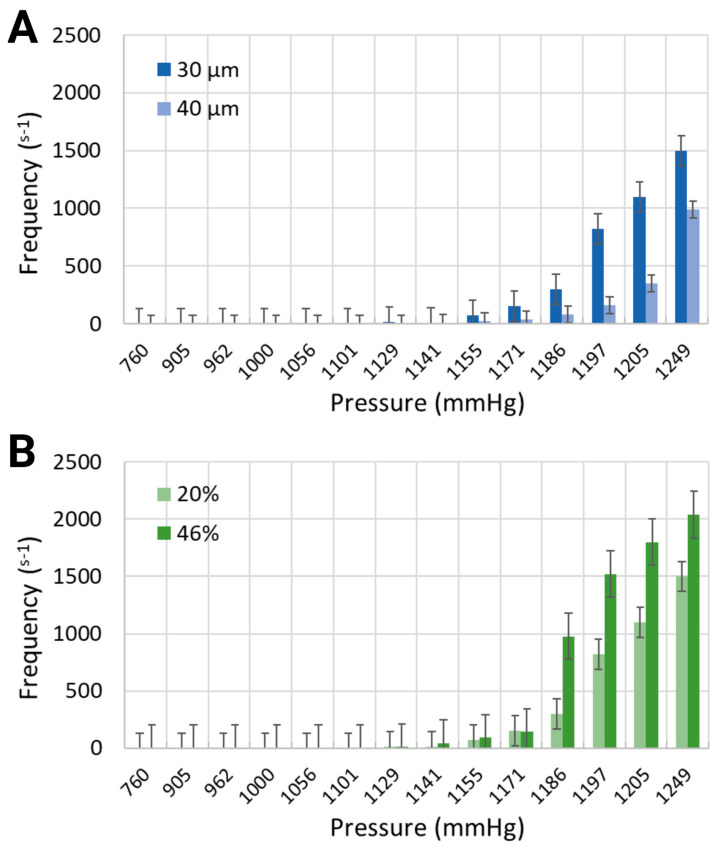
Generation frequency for (**A**) 30 μm and 40 μm channel widths containing the working fluid with 20% equivalent hematocrit concentration, and (**B**) 30 μm channel width containing two working fluids at 20% and 46% hematocrit concentrations.

Various output parameters modulating the formation of intravascular bubbles were studied, specifically two blood-mimicking solutions, i.e., anemia-like 20% [48], and normal-like 46% equivalent hematocrit concentrations, and two geometries, namely channel widths of 30 μm and 40 μm. The observed output parameters of air bubbles consisted of the nucleation density, i.e., the locations of in situ bubble generation per mm and the generation frequency, i.e., the number of formed bubbles per second, following the application of pressure in the range of 760 mmHg to 1248.6 mmHg, due to air injections of 1 mL to 20 mL into the bilateral pressure chambers (results presented in Figure 3 and Figure 4).

As intuitively expected, the intensity of bubble generation increased with the increase in gas pressure in the side channels (Figure 3A,B). However, less expected was that lower equivalent hematocrit concentrations, mimicking lower blood viscosities prevalent in anemic patients [48], and larger channel widths, resulted in a lower nucleation density. For the synthetic blood solution at 20% hematocrit level, local pressures of 1132.4 mmHg and 1193.2 mmHg led to the formation of bubbles with nucleation densities of 0.33 and 6, respectively. This is significantly smaller than for the synthetic blood solution at 46% hematocrit level, which is the value for healthy people, where the same level of pressure application generated bubbles with 0.17 and 14.33 nucleation densities, respectively, within the 30 μm channels.

When the channel width increased from 30 μm to 40 μm while using the same blood-mimicking solution at 20% equivalent hematocrit concentration, the nucleation density exhibited an upward trend for increasing volumes of air injection, but it remained smaller in comparison with the smaller channel width. At 1170.4 mmHg and 1208.4 mmHg, the bubbles emerged with nucleation densities of 1.33 and 8.33 for the 30 μm channel, in comparison with 0.83 and 3.17 for the 40 μm channel.

Regarding the spatial distribution of nucleation sites, Figure 3C depicts the distance between the inlet of the synthetic blood solution and the points of bubble formation. The distribution of bubbles was assessed along the visible 2 mm segment of the vascular channel. For the 30 μm channel width with the synthetic blood solution at 20% equivalent hematocrit concentration, nucleation site distances ranged from 0.28 mm to 1.99 mm. In the 40 μm channels with the same solution, distances spanned from 0.19 mm to 2.00 mm. Meanwhile, for the 30 μm channels with 46% hematocrit solution, nucleation sites were between 0.26 mm and 1.80 mm from the synthetic blood inlet. Notably, the location of nucleation sites was largely independent of the flow direction—whether upstream or downstream.

Similarly to the response of nucleation density to increased air pressure, the frequency of bubble generation also increased (Figure 4). Furthermore, the frequency of bubble generation increased noticeably for channels with smaller widths. This trend was further marginally amplified for the artificial blood with higher equivalent hematocrit concentration. Injected air volumes corresponding to pressures of 1185.6 mmHg and 1193.2 mmHg resulted in generation frequencies of 297.5 s^−1^ and 820 s^−1^ for the synthetic blood solution at 20% hematocrit level, and 976.25 s^−1^ and 1521 s^−1^ for the synthetic blood solution at 46% hematocrit level (30 μm channel width, Figure 4B). The same pressure levels led to respective frequencies of 80 s^−1^ and 162.5 s^−1^ in the 40 μm channels, holding a blood-mimicking solution at 20% equivalent hematocrit concentration (Figure 4A).

### 3.4. Parameters of the Bubbles

Once the simulated gas embolism is initiated, the behavior of the generated bubbles varied depending on several input parameters. The intravascular bubbles were first evaluated for two different widths, i.e., 30 μm and 40 μm, containing a synthetic blood solution at 20% hematocrit level. The bubble characteristics were then assessed for the same channel width, namely 30 μm, containing 20% and 46% equivalent hematocrit concentrations.

**Figure 5:**
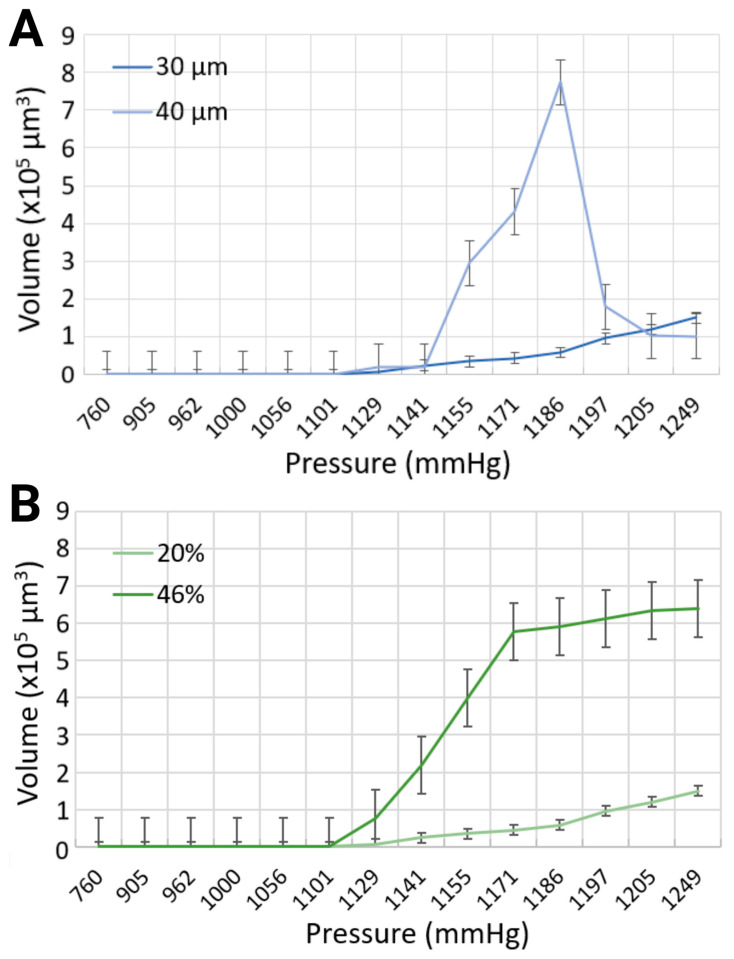
Comparison of maximum bubble volumes in (**A**) 30 μm and 40 μm channels with 20% blood-mimicking solution, and (**B**) 30 μm channel width with 20% and 46% blood-mimicking solutions.

The volume of the bubbles varied substantially, as modulated by the widths of the channels with the same synthetic blood solution, due to (i) simultaneous formation of small and large microbubbles, and (ii) recurring, yet stochastic, coalescence. The maximum bubble volumes were compared for each input parameter at different levels of compression, as presented in Figure 5A. In the 30 μm channels, the largest bubble volume was 150 × 10^3^ μm^3^, corresponding to a length of 125 μm for the 20% equivalent hematocrit concentration, and 640.65 × 10^3^ μm^3^ with a bubble length of 533.87 μm for the 46% equivalent hematocrit concentration, at the same injected air volume of 20 mL, resulting in the highest level of pressure application at 1248.6 mmHg. To put things in perspective, this volume translates in bubbles approximately 2.5 times, and 18 times longer, respectively, than the width of the mimicked blood vessel. However, the maximum bubble volume generated in the 40 μm channel width with a synthetic blood solution at 20% hematocrit level was determined at 773.28 × 10^3^ μm^3^, corresponding to a bubble length of 483.29 μm, for a pressure of 1185.6 mmHg (approximately 10 times longer than the vasculature diameter). As the volume of air injection increased, the bubble volume began to progressively decline until complete dissipation. This counterintuitive observation can be explained, as before by the authors of [15], by the chaotic character of the distribution of bubble sizes at lower pressures, when a smaller number of bubbles are produced, with few large ones, as opposed to the normal distribution at higher pressures, when many, but small, bubbles are present. Although the working fluid with a higher viscosity exhibited larger bubble volumes, the channel width had a more pronounced effect on bubble dimensions (Figure 5B). This effect was particularly evident at the pressure of 1185.6 mmHg in the 40 μm channel, where the bubble volume reached its maximum. The surge in bubble size observed in this case can be attributed to the peak of bubble coalescence, where multiple smaller bubbles fused to form a significantly larger single bubble. This suggests that higher pressures are not necessarily the most hazardous conditions for gas embolism. In fact, lower pressures can also pose significant risks, as they may lead to the stochastic formation of numerous small bubbles, with the occasional appearance of much larger bubbles due to coalescence. This dynamic behavior warrants further investigation in future studies.

**Figure 6:**
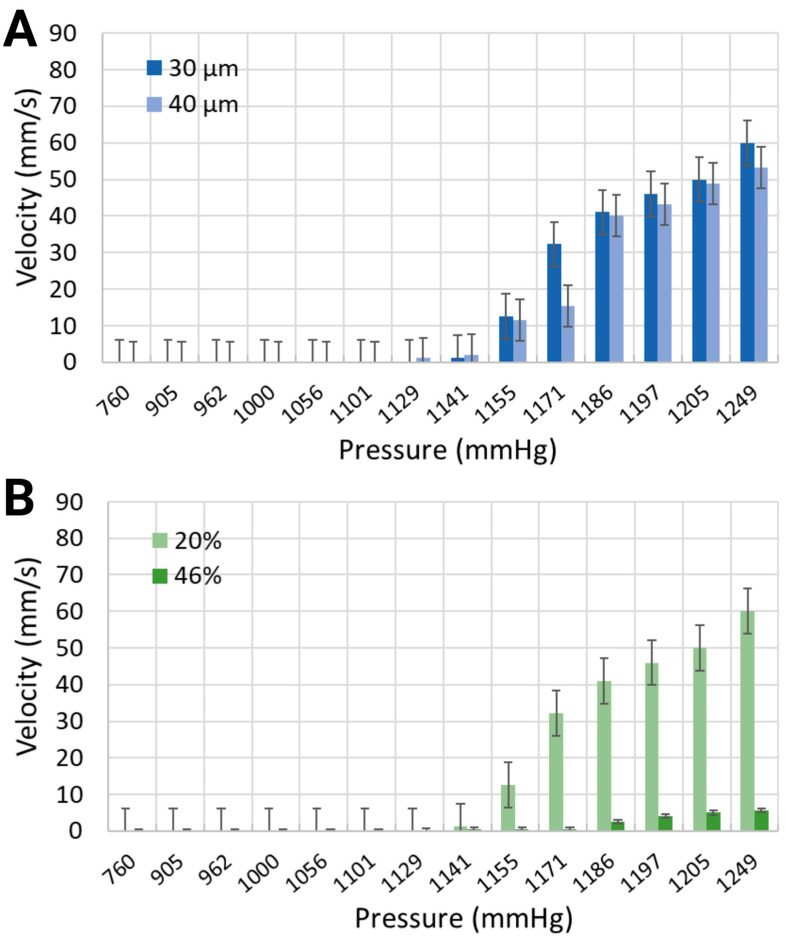
Mean velocity of the synthetic blood solutions in (**A**) two different channel widths with 20% equivalent hematocrit concentrations, and (**B**) the same channel width with 20% and 46% equivalent hematocrit concentrations.

The mean velocity of generated microbubbles in 30 μm channels was larger for the synthetic blood solution at 20% hematocrit level than for the synthetic blood solution at 46% hematocrit level (Figure 6). For local pressure levels of 1170.4 mmHg, 1185.6 mmHg, and 1193.2 mmHg, the average velocities were 32.26 mm/s, 41.02 mm/s, and 45.97 mm/s for the 20% equivalent hematocrit concentration, as opposed to 0.46 mm/s, 2.5 mm/s, and 4.73 mm/s for the 46% equivalent hematocrit concentration. Working fluids with smaller viscosities are typically associated with reduced resistance to flow, thereby enabling bubble motion at higher speeds [49]. The 40 μm width channels, containing a synthetic blood solution at 20% hematocrit level, exhibited slightly slower bubble motion than smaller channel widths. The mean velocities were 15.42 mm/s, 40.15 mm/s, and 43.21 mm/s for the same applied pressures.

### 3.5. Mechanism of Gas Embolism Modulated by Pressure, Width, and Blood Viscosity

The comparative dynamics of gas embolism, modulated by applied pressures ranging from a minimum of 960 mmHg to 1248.6 mmHg (200 mmHg to 488.6 mmHg above the atmospheric pressure of 760 mmHg), were studied and compared in microfluidic systems with 50 μm separations. These systems featured channel widths of 30 μm and 40 μm (for equivalent hematocrit concentrations of 20%), and blood concentrations of 20% and 46% (for channels with 30 μm widths). The dynamics of gas embolism mimicked in vitro can be summarized as follows:

***Dynamics of gas embolism modulated by blood viscosity*** (Movie S1 and Movie S2 in Supplementary Information). In channels with 30 μm widths and 20% equivalent hematocrit concentration (Movie S1), at 370 mmHg above normal atmospheric conditions, very few small, spherical bubbles formed at specific locations but were quickly washed away by the working fluid. A slight increase in pressure, e.g., 380–410 mmHg, resulted in substantial changes in bubble formation dynamics, with higher nucleation densities and larger bubbles that frequently coalesced, forming cylindrical Taylor bubbles that moved rapidly with the fluid. Further increases in pressure, up to 440 mmHg, led to the emergence of two distinct populations of bubbles: large, cylindrical bubbles filling the channels, and small, spherical ones not reaching the walls.

Conversely, in channels with 30 μm widths and 46% hematocrit concentration (Movie S2), the dynamics were more complex. At lower pressures, between 340 mmHg and 370 mmHg, more bubbles formed compared to the lower viscosity blood, but these bubbles grew slowly and were often static. As the pressure increased to 420–430 mmHg, the nucleation density rose, and the bubbles coalesced, forming long cylindrical bubbles that occupied the entire channel. Interestingly, although Taylor bubbles filled most of the observed vessel length, short sections filled with liquid were intermittently observed, with these liquid-filled regions still containing multiple active sites of bubble formation.

***Dynamics of gas embolism modulated by vessel width*** (Movie S1 and Movie S3 in Supplementary Information). The dynamics of gas embolism in vitro in channels with 30 μm widths and 20% hematocrit concentration was previewed above (Movie S1). At small pressures, i.e., 200 mmHg to 360 mmHg, only minor differences were observed compared to the dynamics in narrower channels. However, with a small increase in pressure, i.e., 400 mmHg to 420mm Hg, numerous long Taylor bubbles were observed. These bubbles collected all the smaller bubbles that formed in between, before they were fully grown, thus cascading into even longer bubbles. At higher pressures, i.e., 445 mmHg, the flow transitioned to a fast and biphasic regime. In this state, the long bubbles developed chaotic shapes that did not fully occupy the channel width, allowing blood to flow along the sides.

***Mechanism of Gas Embolism***. The analysis of the videos that recorded gas embolism events in vitro for systems where pressure is applied laterally on the microvasculature with thin walls, i.e., 50 μm or less, suggests that the mechanism of gas transfer for the pressure area to the blood is based on percolation, rather than diffusion. Indeed, in all instances, that is, different blood viscosities and different blood vessel diameters, the nucleation spots, once established, remain constantly fixed in the same position. The percolation rather than diffusion through PDMS further suggests that the microfluidics system developed here has more similarity to blood vessel tissue, which is inherently heterogenous, i.e., comprising cell ‘islets’ and interconnective matter.

## 4. Discussion

The experimental findings can provide insights into the formation and behavior of gas bubbles in biomimetic vascular models, advancing our understanding of gas embolism. By mimicking the structural and rheological properties of blood vessels and blood, respectively, these models can shed light onto key factors that influence bubble nucleation, growth, and movement in physiological contexts.

***Mechanism of Gas Transport at the Microscale***. In general, two mechanisms are known to be responsible for gas embolism: the direct injection of gas in the bloodstream, either intentional, e.g., for ultrasound imaging, or unintentional, e.g., incorrect catheterization; and gas absorption in the blood at high pressure, followed by sudden desorption due to rapid decompression, e.g., in decompression sickness. While these two alternative transport mechanisms are usually considered at the macroscale, they equally require validation at the microscale. For instance, in principle, a compression/decompression cycle of external pressure outside an individual alveolus, especially if sudden, e.g., explosions, airbag release, and forceful resuscitation, could lead to in situ desorption or to ‘direct injection’ if the thin alveolar walls are mechanically compromised. Moreover, the usage of pressured gas in modern medical practice, e.g., laparoscopy, could lead to the exposure of fine blood vessels to higher pressures and thus indirect gas transport in blood, or alternatively to blood vessel damage and thus direct injection. Indeed, it was recently reported [50] that half of the young patients, on which laparoscopic appendectomy was performed, presented gas embolism, occurring during the dissection and ligation of the mesoappendix.

Considering the adsorption/desorption cycle, the difference in pressure in the fluid circuit is very small, i.e., a maximum of 10 mmHg (for the length of the microvasculature exposed to parallel pressure chambers, Table 1). Conversely, the visual analysis of the videos that recorded the gas embolism-like events in vitro for systems where pressure was applied laterally on the microvasculature with thin walls, i.e., 50 μm or less, suggests a different mechanism than the absorption/desorption mechanism, as the spatial distribution of the nucleation of the bubbles did not show any correlation with the—admittedly small—gradient of pressure along the fluid flow. Furthermore, the analysis of the nucleation suggests that the mechanism of gas transfer for the pressure area to the blood is based on percolation, rather than diffusion. Indeed, in all instances, that is, different blood viscosities and different blood vessels diameters, the nucleation spots, once established, remain constantly fixed in the same position. The percolation rather than diffusion through PDMS further suggests that the microfluidic system developed here has more similarity to blood vessels tissue, which is inherently heterogenous, i.e., comprising cell ‘islets’ and interconnective matter.

***Proximity of pressure application.*** The observation that the generation of gas bubbles does not occur at a separation of 100 μm between the source of pressure and blood flow, but occurs readily at 50 μm, highlights the critical role of membrane thickness in gas transport through the walls of blood vessels, even under moderate pressure. These findings underscore the physiological relevance of mimicking the vascular dimensions closely tied to gas transport in the human body, where most blood vessels lie within a 100 μm range from the airways [46,47]. Such spatial constraints could influence embolism risks in clinical scenarios involving rapid pressure changes, such as decompression sickness or hyperbaric exposures, by altering the conditions under which gas bubbles form and propagate. The closer proximity of pressure sources to blood vessels (i.e., within 50 μm), overwhelmingly prevalent in lung tissues, can significantly increase the likelihood of bubble formation, thereby intensifying the risks of pulmonary embolism in environments with fluctuating pressures, such as airbags deployed in car accidents, strong CPR, and explosions.

***Rheological and geometric factors.*** The present study demonstrated that blood viscosity and vascular geometry significantly impact the formation and behavior of intravascular microbubbles. Higher hematocrit levels, associated with increased blood viscosity, were linked to greater nucleation densities, higher bubble frequencies, and larger bubbles. These findings suggest that increased viscosity exacerbates gas embolism risks by promoting conditions favorable for bubble formation and persistence. This observation is particularly relevant to men, who typically exhibit higher hematocrit levels than women [51], contributing to their greater susceptibility to gas embolism. On the other hand, a 20% equivalent hematocrit concentration, as seen in conditions like anemia, resulted in lower nucleation densities and smaller bubbles, reflecting the reduced blood viscosity, and therefore the decreased resistance to flow. The latter causes lower pressure drops in the synthetic blood solution, which, in turn, creates smaller pressure gradients than those generated in higher blood viscosities, as global pressures are applied in the vicinity of the vascular channel. While this may suggest a lower risk of embolism in anemic individuals under normal conditions, the altered flow dynamics and bubble behavior in low-viscosity environments could still pose unique risks under specific circumstances.

Furthermore, larger widths of blood vessels, e.g., arteries, facilitated larger bubble volumes, likely due to reduced flow resistance and increased energy availability for bubble nucleation and growth. This trend underscores the higher risk of gas embolism in the arterial system compared to the venous one, where smaller widths and slower flow rates limit bubble dynamics. However, smaller channel widths were found to be more vulnerable to bubble formation, due to increased nucleation densities. This vulnerability is consistent with findings in studies of decompression sickness, where researchers have reported that gas embolism is believed to originate in veins [52], where less energy is required for bubble nucleation to occur, due to blood circulation being at lower flow rates than in the arteries.

***Dynamic behavior of bubbles.*** Bubble volume and velocity also varied significantly with changes in channel geometry and fluid properties. Smaller channels and lower viscosities allowed faster bubble movement, consistent with reduced resistance to flow. Conversely, higher viscosities, driven by increased hematocrit levels, produced slower-moving, larger bubbles due to enhanced coalescence and resistance. Such bubbles pose a greater obstruction risk, particularly in arterial systems, where the combination of low velocity and large vessel size exacerbates their impact on blood circulation.

One important qualification with respect to the present study is the absence of red blood cells in the current experimental setup. In vivo, red blood cells are believed to contribute to bubble lodging due to the formation of blood clots at the tail of the bubble [16,17,18]. The present study suggests that future research should incorporate these cells into the experimental setup to enhance the clinical relevance of these findings.

***Clinical relevance and implications.*** The experimental conditions modeled in these biomimetic devices provide parallels to physiological conditions that can trigger and then precipitate gas embolism. The pronounced effects of higher hematocrit levels and larger vascular channels help explain sex-specific differences in embolism susceptibility, as well as the heightened severity of arterial gas embolism compared to venous embolism. Arterial embolisms are particularly severe because bubbles are more likely to lodge in critical pathways, obstructing blood flow and causing tissue damage. In contrast, venous embolisms often encounter the pulmonary bubble filter [53], which mitigates their progression.

By providing a controlled environment to simulate and study gas bubble formation and behavior, these biomimetic devices offer a powerful tool for exploring gas embolism mechanisms. They pave the way for targeted therapeutic interventions, such as modifying blood viscosity or designing medical protocols to minimize embolism risks under specific physiological or procedural conditions. Future studies incorporating red blood cells and clotting dynamics will further refine these findings and enhance their applicability to clinical scenarios.

## 5. Conclusions

The present study aims to contribute to the fundamental understanding of the genesis of vascular gas embolism for the mitigation of bubble formation, through the design and use of a novel microfluidic design monitoring the impact of local pressure applications. Although microfluidic devices are routinely used for diagnostics and high-throughput screening, they are emerging as invaluable tools for studies mimicking in vitro biological process at the microscale, such as—here—gas embolism, thereby advancing scientific and medical knowledge. The proposed biomimetic platforms allowed the simulation of a controllable environment, with the ability to fine-tune experimental conditions in order to dissect the underlying mechanisms governing intravascular bubble formation. Microscopic-level information can be obtained and analyzed in increasingly complex vascular systems on a chip in order to derive adequate diagnostic, preventive, and therapeutic techniques while providing a better understanding of the medical implications of microbubbles on the vascular system. This approach can also be translated into design guidelines for surgical instruments and medical interventions, which can prevent the application of forces on human tissue that lead to gas embolism. Furthermore, the use of bilateral pressure chambers provides insights into the relationship between local pressure variations and the emergence of bubbles within vascular networks. Geometric variations and blood viscosities were proven to have a considerable impact on the genesis and evolution of intravascular microbubbles. The tendency of microbubbles to coalesce and form larger, slower-moving bubbles in thicker blood-mimicking solutions and larger channel widths was supported by empirical-based evidence from the current findings.

## Figures and Tables

**Table 1:** Calculation of pressure drops, using the Hagen–Poiseuille equation for fluid flow in a rectangular channel, for the region of interest (ROI), i.e., 6 mm, and the full length of the microfluidic channels.

Channel Width (μm)	Hematocrit Concentration (%)	Total Pressure Drop (mmHg)	ROI Pressure Drop (mmHg)
30	20	10.43	3.91
40	20	13.67	5.13
30	46	25.03	9.38

## Data Availability

The raw data supporting the conclusions of this article will be made available by the authors on request.
